# Novel blended catalysts consisting of a TiO_2_ photocatalyst and an Al_2_O_3_ supported Pd–Au bimetallic catalyst for direct dehydrogenative cross-coupling between arenes and tetrahydrofuran†

**DOI:** 10.1039/c8ra02948b

**Published:** 2018-07-02

**Authors:** Akanksha Tyagi, Akira Yamamoto, Hisao Yoshida

**Affiliations:** Graduate School of Human and Environmental Studies, Kyoto University, Yoshida Nihonmatsu-cho Sakyo-ku Kyoto 606-8501 Japan yoshida.hisao.2a@kyoto-u.ac.jp; Elements Strategy Initiative for Catalysts and Batteries (ESICB), Kyoto University, Kyotodaigaku-Katsura Nishikyo-ku Kyoto 615-8520 Japan yoshida.hisao.2a@kyoto-u.ac.jp

## Abstract

Dehydrogenative cross-coupling (DCC) is a clean methodology to make C–C bonds by using abundant C–H bonds. The blended catalyst, developed in this study, consists of a TiO_2_ photocatalyst and an Al_2_O_3_ supported Pd–Au bimetallic catalyst and shows superior activity to the conventional TiO_2_ photocatalyst loaded with the corresponding metal co-catalyst for the direct DCC between various arenes and tetrahydrofuran, with concomitant evolution of hydrogen gas. The reactions were done under mild conditions without consuming any oxidising agent or other additional chemicals. This new approach of separating the photocatalyst and the metal catalyst parts allows their independent modification to improve the overall catalytic performance.

## Abstract 2

A TiO_2_ photocatalyst physically mixed with a supported Pd–Au bimetallic catalyst is more efficient than Pd loaded TiO_2_ sample for the photocatalytic DCC between arene and THF.

## Graphical abstract

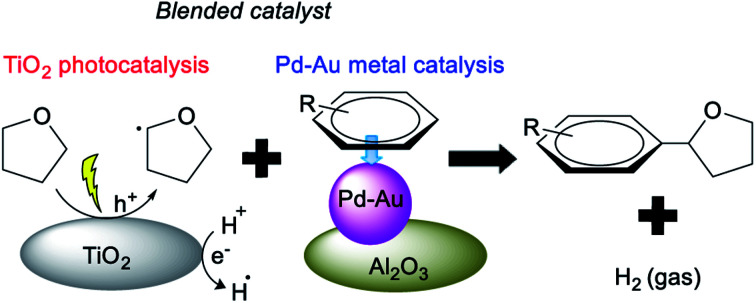

## Introduction

Dehydrogenative cross-coupling (DCC) is an efficient methodology to make carbon–carbon (C–C) bonds between two different organic compounds.^1^ This direct route to make C–C bonds yields hydrogen as a by-product, which can be used as a fuel or for hydrogenation reactions. Different catalysts have been developed to carry out the thermal dehydrogenation reactions,^2^ but the scope for further improvement remains.

Recently, the use of an abundant, safe, and heterogeneous TiO_2_ photocatalyst for DCC has become more attractive as it works under mild reaction conditions.^3^ The photogenerated holes on TiO_2_ can activate the C–H bonds in various organic molecules to generate the corresponding radical species and protons. The generated radicals can further react with other radical species or molecules to give the coupling products by making new C–C bonds, while the protons are reduced by the photoexcited electrons to form hydrogen molecules. Thus, the TiO_2_ photocatalyst efficiently brings out DCC with concomitant hydrogen gas evolution, without any external oxidising agent. In many cases, metal nanoparticles are loaded on TiO_2_ (M/TiO_2_), that can accept the excited electrons from the TiO_2_ photocatalyst, to decrease the electron–hole recombination and increase the photocatalytic efficiency.^4^ In these cases, the metal nanoparticles function as an electron receiver.

On the other hand, it is notable that the metal nanoparticles loaded on TiO_2_ sometimes can also participate catalytically in some photocatalytic reactions, thus acting as co-catalysts.^3*b*–3*e*,5^ For example, Pd nanoparticles catalyse a reaction between an aromatic molecule and different carbon centred radicals,^3*b*–3*d*^ and Pt nanoparticles catalyse reactions like photocatalytic hydrogenation,^5*a*^ dehydrogenation,^5*c*^ and C–H bond activation.^3*e*,5*b*^ To develop such a hybrid catalyst system consisting of a photocatalyst and a metal co-catalyst, one may plan some modifications to improve the catalytic activity, *e.g*., modifying the metal co-catalyst by increasing its loading amount and dispersion, making an alloy with other metal, varying its oxidation state and so on. However, it is difficult to realise these alternations of the metal co-catalyst part on the TiO_2_ surface without changing the photocatalytic performance of the TiO_2_ part. It is because TiO_2_ itself is susceptible to chemical or thermal treatments and its physical properties like surface area, crystal phase, and crystal defects, may be varied.^6^

Here, a novel blended catalyst is proposed as one solution to this problem, which is a physical mixture of a TiO_2_ photocatalyst and a supported metal catalyst. Since the two catalysts can be prepared separately without affecting each other, they can be modified independently to achieve high activity entirely. Although several reports mentioned the use of a kind of the blended catalyst including a photocatalyst,^7^ they were employed just for investigating the origin of the catalytic activity. Employing a blended catalyst to improve the activity is uncommon, except for a few examples, such as a combination of two kinds photocatalysts to build a Z-scheme for water splitting,^8*a*–*c*^ and a combination of a photocatalyst and an acid catalyst for C–O coupling.^8*d*^ Thus, this is the first report for the blended catalyst consisting of a photocatalyst and a metal catalyst for the enhancement of the catalytic activity. We have developed it for the sp^2^C–sp^3^C photocatalytic direct DCC between various arenes and ethers like tetrahydrofuran (THF). In this study, we found that a blended catalyst consisting of a TiO_2_ photocatalyst and an Al_2_O_3_ supported Pd–Au bimetallic catalyst can convert various arenes and THF to their corresponding DCC products and hydrogen, with higher activity than the conventional metal loaded TiO_2_ photocatalyst, under mild conditions without consuming any oxidising agent or other chemicals.

## Experimental section

### Catalyst preparation

The Pd loaded TiO_2_ sample (Pd(3.0)/TiO_2_) (the number in parenthesis refers to the loading amount of the metal in weight%), and the bimetallic Pd–Au loaded TiO_2_ sample (Pd(2.0)Au(1.0)/TiO_2_) was prepared by a photodeposition method as follows. For the Pd(3.0)/TiO_2_ sample, 4 g of TiO_2_ powder (JRC-TIO-8 provided by Catalysis Society of Japan, anatase phase, surface area 335 m^2^ g^−1^) was dispersed in ion-exchanged water (300 mL) and irradiated for 30 min from a ceramic xenon lamp (PE300BUV). Then, 100 mL methanol and 5.8 mL of an aqueous solution of PdCl_2_ (10.1 mg mL^−1^ of Pd) was added to the suspension and the contents were stirred for 15 min without irradiation, followed by 1 h stirring under the light irradiation. It was then filtered off with suction, washed with ion-exchanged water, and dried at 323 K for 12 h to get the Pd(3.0)/TiO_2_ sample. The bimetallic Pd(2.0)Au(1.0)/TiO_2_ sample was prepared in a similar manner by using 2 g TiO_2_, 175 mL water, 50 mL methanol, 6.5 mL of an aqueous solution of PdCl_2_ (6.12 mg mL^−1^ of Pd), and 4.1 mL of an aqueous solution of HAuCl_4_ (4.8 mg mL^−1^ of Au).

The various Al_2_O_3_ supported samples (monometallic Pd(3.0)/Al_2_O_3_ and Au(3.0)/Al_2_O_3_ and bimetallic Pd(*x*)Au(*y*)/Al_2_O_3_) were prepared in a similar manner to the reported method^9^ with some modifications, as mentioned below. 2 g of the Al_2_O_3_ powder (JRC-ALO-7 provided by the Catalysis Society of Japan, γ-phase, 180 m^2^ g^−1^) and the desired volume of the aqueous solutions of PdCl_2_ and HAuCl_4_ were dispersed in 60 mL of ion-exchanged water. The suspension was vigorously stirred at room temperature for 15 min. The pH of the suspension was adjusted to 10 by using an aqueous solution of NaOH (1 M). The resultant slurry was stirred for 24 h at room temperature. Then, the contents were filtered and dried overnight in an electric oven at 323 K. Finally, the powder was reduced at 423 K under pure H_2_ for 30 min to get the Pd(*x*)Au(*y*)/Al_2_O_3_ samples. The total loading amount of the metals on the support was fixed to 3 weight%. For comparison, the monometallic Al_2_O_3_-supported samples, Pd(3.0)/Al_2_O_3_ and Au(3.0)/Al_2_O_3_, were also prepared by a similar procedure.

The prepared samples were characterised by various techniques like TEM, XRD, XAFS, and UV-DRS. The transmission electron microscopy (TEM) images of the various samples were taken by a JEM-2200FS Field Electron Microscope. X-ray diffraction (XRD) measurements of the Al_2_O_3_ support and various Al_2_O_3_ supported samples were carried out on a Lab X XRD-6000 Shimadzu. X-ray absorption fine structure (XAFS) was used to study the electronic state of the Pd and Au species in the Pd(*x*)Au(*y*)/Al_2_O_3_ samples. The Pd K-edge and Au L_III_-edge XAFS measurements were carried out at the BL01B1 beam line of the synchrotron facility at Spring-8 in the RIKEN Harima institute (Hyogo, Japan). The measurements were done in a transmission mode. The samples were mixed with a calculated amount of boron nitride, and the mixture was shaped into a pellet which was used for measurements. The spectra were analysed by Athena software.^10^ The ultraviolet-visible diffuse reflectance spectroscopy (UV-DRS) measurements of the monometallic Pd(3.0)/Al_2_O_3_ and Au(3.0)/Al_2_O_3_ samples, and the bimetallic Pd(*x*)Au(*y*)/Al_2_O_3_ samples were carried out on a JASCO V-570 instrument.

### Procedure for the photocatalytic activity tests

All chemicals were of analytical grade and used without further purification; THF (Wako Pure Chemicals, 99%), benzene (Nacalai Tesque, 99%), benzaldehyde (Wako Pure Chemicals, 99%), benzonitrile (Kishida Chemicals, 99%), toluene (Nacalai Tesque, 99%), and aniline (Kishida Chemicals, 99.5%). The cross-coupling product 4-(tetrahydrofuran-2-yl)benzonitrile was synthesised according to the literature^11^ (details are mentioned in the ESI†).

The sp^2^C–sp^3^C photocatalytic direct DCC reactions between different arenes and THF were carried out in a closed reactor of a Pyrex test tube (volume = 80 mL) and the xenon lamp (PE300BUV) as the light source. The wavelength of the irradiated light was restricted to ≥350 nm, by using a long pass optical filter. The light intensity was maintained at 40 mW cm^−2^ (measured at 360 nm ± 60 nm). The reaction test involved the pre-treatment of the catalyst (photocatalyst or the blended catalyst) by light irradiation from the xenon lamp for 1 h. Next, the test tube was sealed with a silicon septum and after a 10 min argon purge, the reactants (arene and THF) were added and the resultant suspension was magnetically stirred under the light irradiation for the desired time. After the reaction, a part of the gaseous phase was collected in an air-tight syringe and analysed by GC-TCD (Shimadzu, GC-8A). The liquid phase was first diluted by ethanol (2 mL), filtered, and analysed by GC-MS (Shimadzu, GCMS-QP5050A) by using decane as an internal standard. The amount of all DCC products was approximately determined from the calibration curve of 4-(tetrahydrofuran-2-yl)benzonitrile. The GC-MS yield of the products (% *Y*) was calculated as % *Y* = 100 × [total amount of the DCC products (μmol)/initial amount of the arene (μmol)]. The selectivity to the DCC products, based on the arene, (% *S*) was calculated as % *S* = 100 × [total amount of DCC product (μmol)/total amount of the products from arene (μmol)].

## Results and discussion

### **Characterisation** of Pd(*x*)Au(*y*)/Al_2_O_3_ samples

**Fig. 1:**
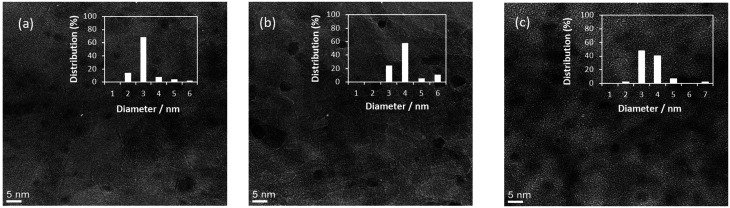
TEM images and particle distribution histograms of (a) monometallic Pd(3.0)/Al_2_O_3_, (b) Au(3.0)/Al_2_O_3_, and (c) bimetallic Pd(2.0)Au(1.0)/Al_2_O_3_ samples.

Fig. 1 shows the TEM images and particle distribution histograms of three Al_2_O_3_ supported samples, monometallic Pd(3.0)/Al_2_O_3_ (Fig. 1a), monometallic Au(3.0)/Al_2_O_3_ (Fig. 1b), and bimetallic Pd(2.0)Au(1.0)/Al_2_O_3_ (Fig. 1c) that exhibited the highest catalytic activity as mentioned later (Pd/Au molar ratio: 3.5). Clearly, all samples contained small and well-dispersed nanoparticles of 3–4 nm in size.

**Fig. 2:**
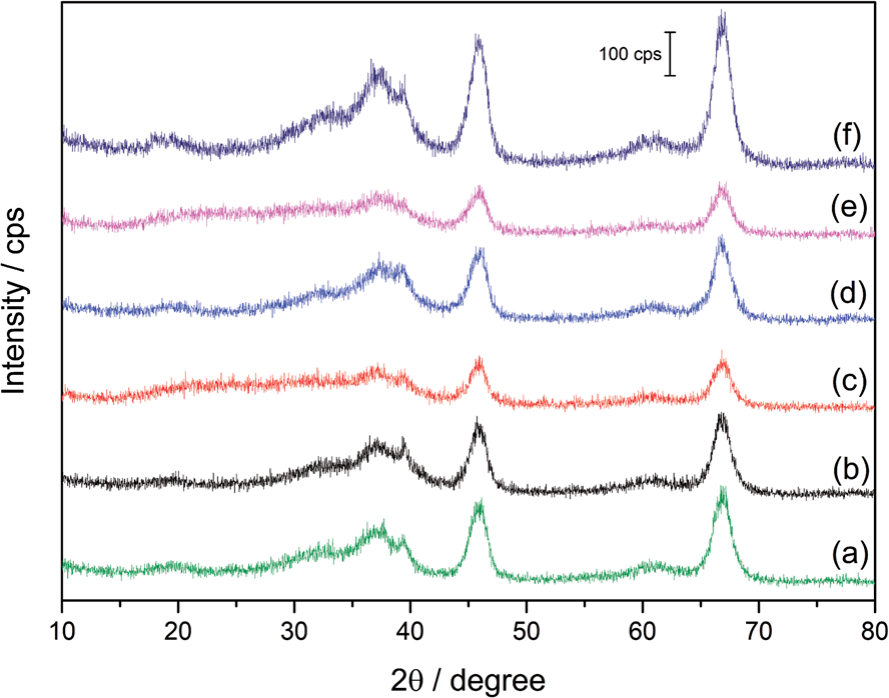
XRD patterns of the support, the monometallic and the bimetallic samples: (a) Al_2_O_3_, (b) Pd(3.0)/Al_2_O_3_, (c) Pd(2.5)Au(0.5)/Al_2_O_3_, (d) Pd(1.5)Au(1.5)/Al_2_O_3_, (e) Pd(0.5)Au(2.5)/Al_2_O_3_, and (f) Au(3.0)/Al_2_O_3_.

Fig. 2 shows the XRD patterns of the Al_2_O_3_ support (Fig. 2a), the monometallic Pd(3.0)/Al_2_O_3_ (Fig. 2b), monometallic Au(3.0)/Al_2_O_3_ sample (Fig. 2f), and the bimetallic Pd(*x*)Au(*y*)/Al_2_O_3_ samples (Fig. 2c–e). According to literature, the Pd (111), (200), and (220) diffractions should appear at 2*θ* = 40.1°, 46.5°, and 68.1°, respectively (ICSD coll. code 41517) while the Au (111), (220), and (311) diffractions should be at 2*θ* = 38.1°, 64.5°, and 77.1°, respectively (ICSD coll. Code 52 249). However, none of these diffractions were observed in the XRD patterns of the prepared monometallic Pd(3.0)/Al_2_O_3_ and Au(3.0)/Al_2_O_3_, and bimetallic Pd(*x*)Au(*y*)/Al_2_O_3_ samples. This could be due to the small size and high dispersion of the metal nanoparticles, as revealed by TEM. Another reason could be the broad diffractions from the Al_2_O_3_ support around 2*θ* = 38°, 46°, and 67°,^12^ which may have interfered with the very small and broad diffractions from the metal nanoparticles in the prepared samples.

**Fig. 3:**
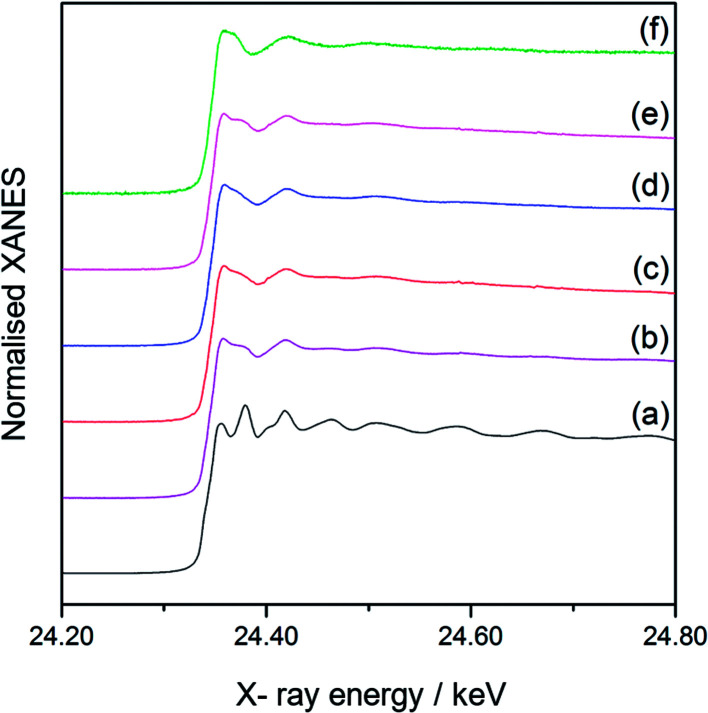
Normalised Pd K-edge XANES of metallic Pd as a reference, and the monometallic and bimetallic catalyst samples: (a) a Pd foil, (b) Pd(3.0)/Al_2_O_3_, (c) Pd(2.5)Au(0.5)/Al_2_O_3_, (d) Pd(2.0)Au(1.0)/Al_2_O_3_, (e) Pd(1.5)Au(1.5)/Al_2_O_3_, and (f) Pd(0.5)Au(2.5)/Al_2_O_3_.

**Fig. 4:**
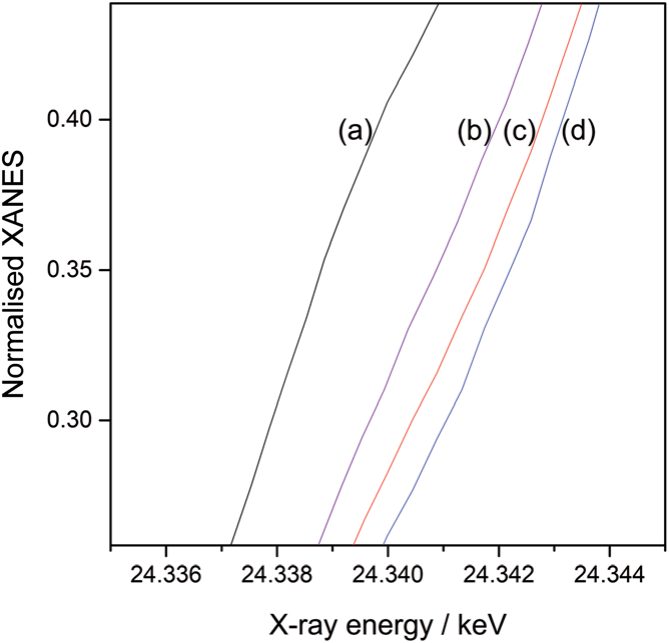
Expanded normalised Pd K-edge XANES of (a) a Pd foil reference foil, (b) the monometallic Pd(3.0)/Al_2_O_3_ sample, and the bimetallic (c) Pd(2.5)Au(0.5)/Al_2_O_3_ and (d) Pd(2.0)Au(1.0)/Al_2_O_3_ samples.

**Fig. 5:**
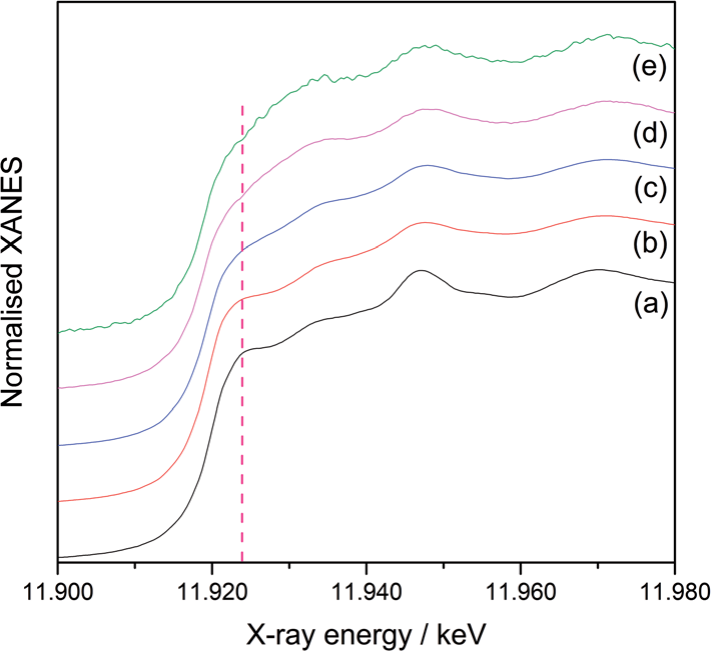
Normalised Au L_III_-edge XANES of a metallic Au as a reference, and the monometallic and bimetallic catalyst samples: (a) an Au foil, (b) the monometallic Au(3.0)/Al_2_O_3_ sample, and the bimetallic (c) Pd(0.5)Au(2.5)/Al_2_O_3_, (d) Pd(1.5)Au(1.5)/Al_2_O_3_, and (e) Pd(2.0)Au(1.0)/Al_2_O_3_ samples.

Fig. 3 shows the normalised Pd K-edge X-ray absorption near edge structures (XANES) of the reference Pd foil (Fig. 3a), the monometallic Pd(3.0)/Al_2_O_3_ sample (Fig. 3b), and the Pd(*x*)Au(*y*)/Al_2_O_3_ samples (Fig. 3c–f). The spectral features in the prepared samples were clearly different from those of the foil, which would mainly originate from the property of the nanoparticles. The samples containing a large amount of Au exhibited slightly different shape from others (Fig. 3f), suggesting the Pd atoms are affected by the Au atoms and have local structure or electronic state different from those in the Pd metal. Also, a clear shift in the absorption edge towards the higher energy was observed in the order: the reference Pd foil (Fig. 4a), the monometallic Pd(3.0)/Al_2_O_3_ sample (Fig. 4b), the bimetallic Pd(2.5)Au(0.5)/Al_2_O_3_ (Fig. 4c) and Pd(2.0)Au(1.0)/Al_2_O_3_ samples (Fig. 4d). The shift from the Pd foil to the monometallic Pd(3.0)/Al_2_O_3_ sample is often observed in the supported metal nanoparticles, and can be attributed to the acidic property of the Al_2_O_3_ support and nanosize of the particles. The further shifts from the monometallic Pd to the bimetallic Pd–Au samples suggest that the Pd species became electron deficient upon the introduction of Au.^13^ This would arise if the Pd and Au atoms in nanoparticles are in intimate contact, and a transfer of electron density occurs from Pd atoms to Au atoms, *i.e.*, Pd^*δ*+^–Au^*δ*−^. Fig. 5 shows the normalised XANES of Au L_III_-edge. The XANES features of the prepared Al_2_O_3_ supported samples, especially at the higher energy region, were similar to that of Au foil indicating that the Au species in these samples was almost metallic. However, the Au absorption edge was clearly shifted in the prepared supported samples at 11 919.7 eV (Fig. 5b–e) from the Au foil at 11 920.2 eV (Fig. 5a), suggesting that the Au atoms became electron rich on the support. A clear absorption shoulder peak was observed around 11 923 eV in the Au foil and the monometallic Au(3.0)/Al_2_O_3_ sample, which corresponds to the transition from the filled core 2*p*_3/2_ level to the vacant d orbitals.^14^ The intensity of this shoulder peak gradually decreased with increasing the Pd content in the bimetallic Pd(*x*)Au(*y*)/Al_2_O_3_ samples, indicating the filling of the d orbitals in Au, which would arise due to the transfer of electron density from Pd to Au atoms, *i.e.*, Pd^*δ*+^–Au^*δ*−^. Thus, the XANES results revealed that the Pd and Au atoms had an intimate contact in the bimetallic Pd(*x*)Au(*y*)/Al_2_O_3_ samples, which affected their local structure and facilitated the electron transfer. As a result, the Pd atom are proposed to be electron deficient in the bimetallic Pd(*x*)Au(*y*)/Al_2_O_3_ samples.

**Fig. 6:**
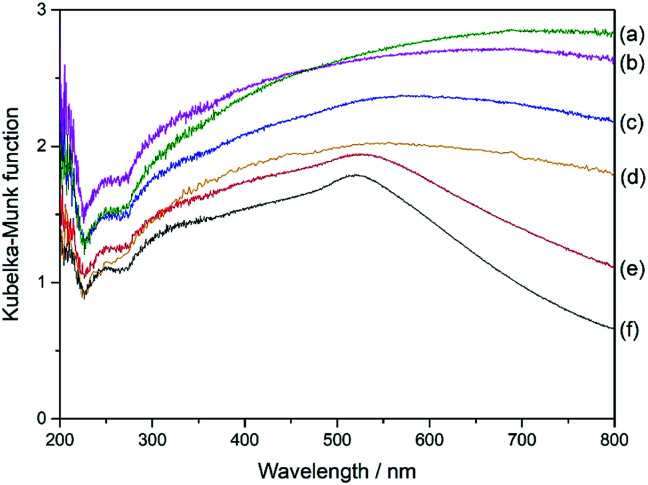
UV-DRS of the Al_2_O_3_-supported samples; (a) Pd(3.0)/Al_2_O_3_, (b) Pd(2.5)Au(0.5)/Al_2_O_3_, (c) Pd(2.0)Au(1.0)/Al_2_O_3_, (d) Pd(1.5)Au(1.5)/Al_2_O_3_, (e) Pd(0.5)Au(2.5)/Al_2_O_3_, and (f) Au(3.0)/Al_2_O_3_.

Fig. 6 shows the UV-DR spectra of different Al_2_O_3_ supported samples. The monometallic Pd(3.0)/Al_2_O_3_ sample showed a very broad band over almost the entire range without any clear obvious peak (Fig. 6a). With the introduction of Au to the samples, the spectra gradually changed (Fig. 6b–e) and the plasmonic peak of Au nanoparticles became clearly visible around 520 nm in wavelength for the Pd(0.5)Au(2.5)/Al_2_O_3_ sample and the monometallic Au(3.0)/Al_2_O_3_ sample (Fig. 6e and f). However, the spectra of the bimetallic samples could not be simulated by that of the two monometallic Pd(3.0)/Al_2_O_3_ and Au(3.0)/Al_2_O_3_ samples, which suggests that the Pd and Au species are not independently present on the surface of the support but in close contact, and thus they affect the electronic properties of each other, which would provide unique catalytic property.

Based on these results we conclude that the Pd and Au atoms in the bimetallic Pd(*x*)Au(*y*)/Al_2_O_3_ samples have a strong interaction, making the Pd atoms to be electron deficient.

### Photocatalytic direct DCC between benzene and THF with different catalysts

Various photocatalysts like TiO_2_, Pd(3.0)/TiO_2_ and Pd(2.0)Au(1.0)/TiO_2_, and Al_2_O_3_ supported metal catalysts like Pd(3.0)/Al_2_O_3_, Au(3.0)/Al_2_O_3_ and Pd(*x*)Au(*y*)/Al_2_O_3_ were used for the photocatalytic direct DCC between arenes and THF.

To begin with, the DCC of benzene (1) and THF was examined with different catalysts (Table 1). The reaction with the Pd(3.0)/TiO_2_ sample gave 2-phenyltetrahydrofuran (1a) as the only DCC product (Table 1 entry 1), along with small amounts of side products like octahydro-2,2′-bifuran, a homocoupling product of THF, and some unknown products (amounts not shown). The gas phase contained hydrogen (Fig. S1†), but, due to these side reactions, the amount of hydrogen and the DCC products was not balanced. Hence, the amount of hydrogen couldn't be determined precisely. The reaction done with a pristine TiO_2_ sample did not produce 1a (Table 1, entry 2), indicating that Pd loading was necessary for the DCC reaction.

**Table 1:** Photocatalytic direct DCC between benzene and THF with various catalysts^a^

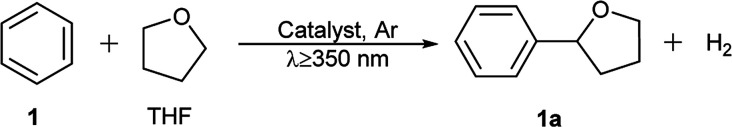			
Entry	Catalyst	1a (μmol)	% *S*
1	Pd(3.0)/TiO_2_	8.9	>99
2	TiO_2_	0.0	n.a.^b^
3	Pd(2.0)Au(1.0)/TiO_2_	1.4	>99
4	TiO_2_ + Pd(3.0)/Al_2_O_3_	11.9	>99
5^c^	Pd(3.0)/Al_2_O_3_	0.0	n.a.

a Reaction conditions: 2 mL (22.4 mmol) of benzene, 2 mL (24 mmol) of THF, 50 mg of photocatalyst or 100 mg of the blended catalyst (50 mg of each the photocatalyst and the metal catalyst) was used, the reaction time was 1 h. The amount of 1a was approximately determined from the calibration curve of 4-(tetrahydrofuran-2-yl)benzonitrile. Other conditions and selectivity calculations are mentioned in the Experimental section.

b Not applicable.

c The reaction was done without the TiO_2_ sample.

Attempts were made to modify the Pd metal part by simultaneous loading Au on the TiO_2_ photocatalyst so as to make bimetallic Pd–Au nanoparticles (Pd(2.0)Au(1.0)/TiO_2_). However, this sample gave much lower yield of 1a (Table 1, entry 3) than the monometallic Pd(3.0)/TiO_2_ sample (Table 1, entry 1). Since this modification was not helpful for the enhancement of catalytic activity, we decided to separate the metal part from the TiO_2_ part and designed a blended catalyst consisting of a TiO_2_ photocatalyst and an Al_2_O_3_ supported metal catalyst.

At the beginning, the reaction was done with a blended catalyst consisting of a pristine TiO_2_ sample and a monometallic Pd(3.0)/Al_2_O_3_ sample (Table 1, entry 4). The reaction gave a much larger amount of 1a and a slightly smaller amount of hydrogen (not shown) than that with the Pd(3.0)/TiO_2_ sample (Table 1, entries 1 and 4). This means that the Pd nanoparticles can contribute to the formation of 1a even deposited on a photo-inactive support like Al_2_O_3_ instead of TiO_2_, or rather work more efficiently on Al_2_O_3_. This result showed that, in this reaction system, the Pd nanoparticles function not just as an electron receiver on the TiO_2_ photocatalyst but also as a catalyst. Moreover, the reaction done with the Pd(3.0)/Al_2_O_3_ sample alone, under light irradiation did not yield 1a (Table 1, entry 5), ruling out the possibility of plasmonic photocatalysis by the Pd metal nanoparticles as reported in some studies,^15^ and confirming that the coexistent TiO_2_ photocatalyst is essential. These results suggest that the formation of 1a is a hybrid of TiO_2_ photocatalysis and Pd metal catalysis. This revelation means that we can modify the property of the two catalytic components independently to improve the product yield. This time we have focused on the modification of the Pd catalyst part, and examined the bimetallic Pd(*x*)Au(*y*)/Al_2_O_3_ catalysts.

### Photocatalytic direct DCC between benzene and THF with a TiO_2_ photocatalyst blended with Pd(*x*)Au(*y*)/Al_2_O_3_ catalysts

Table 2 shows the results of the DCC between benzene and THF with blended catalysts consisting of the TiO_2_ photocatalyst and various bimetallic Pd(*x*)Au(*y*)/Al_2_O_3_ catalysts. When compared to the result with the monometallic Pd(3.0)/Al_2_O_3_ catalyst (Table 1, entry 4), an introduction of a small amount of Au to Pd increased the yield of 1a (Table 2, entries 1 and 2). However, a further increase in the Au content had negative effect on the yield (Table 2, entries 3 and 4) and the monometallic Au(3.0)/Al_2_O_3_ catalyst was inactive for the formation of 1a (Table 2, entry 5). These results indicate that Pd was the catalytically active component of these bimetallic samples. Also, an optimum ratio of Pd and Au is needed for the high activity of the bimetallic samples. This suggests that the increase of the Au content would withdraw the electron density of the active Pd atoms to enhance the reaction, and then the decreasing number of the active Pd sites would decrease the activity. Furthermore, the bimetallic Pd(2.0)Au(1.0)/Al_2_O_3_ catalyst did not yield 1a or H_2_ gas in the absence of the TiO_2_ photocatalyst (Table 2, entry 6), confirming that they could not catalyse the DCC reaction alone and required TiO_2_ photocatalysis. This result also ruled out the possibility of plasmonic catalysis by the bimetallic samples for the production of 1a and H_2_.

**Table 2:** Photocatalytic direct DCC between benzene and THF with the blended catalyst consisting of TiO_2_ photocatalyst and the supported Pd–Au bimetallic catalysts^a^

Entry	Photocatalyst	Al_2_O_3_ supported catalyst	1a (μmol)	% *S*
1	TiO_2_	Pd(2.5)Au(0.5)	13.1	>99
2	TiO_2_	Pd(2.0)Au(1.0)	15.2	>99
3	TiO_2_	Pd(1.5)Au(1.5)	5.8	>99
4	TiO_2_	Pd(0.5)Au(2.5)	2.2	>99
5	TiO_2_	Au(3.0)	0.0	n.a.^b^
6^c^	None	Pd(2.0)Au(1.0)	0.0	n.a.

a All reaction conditions and abbreviations were the same as those described in Table 1.

b Not applicable.

c The reaction was done without the TiO_2_ sample.

### Scope of arene molecule for the photocatalytic direct DCC with THF

**Fig. 7:**
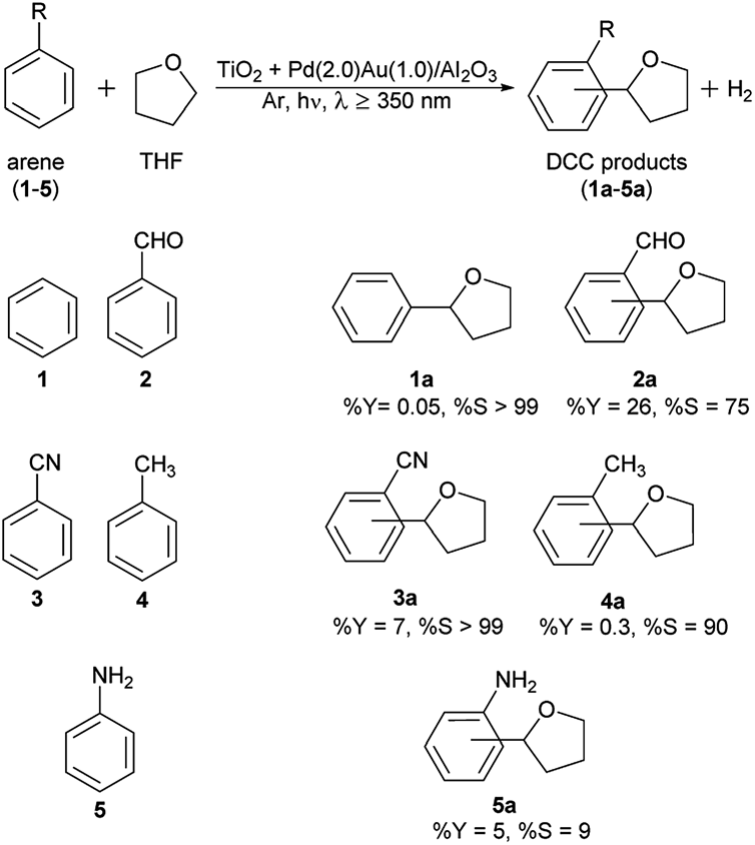
Photocatalytic direct DCC between various arenes and THF with a blended catalyst consisting of a TiO_2_ photocatalyst and a bimetallic Pd(2.0)Au(1.0)/Al_2_O_3_ catalyst. The general reaction conditions and % *S* calculation were the same as Table 1. The definition and calculation of the yield (% *Y*) is mentioned in the Experimental section. Briefly, 50 μL of an arene, 2 mL (24 mmol) of THF, and 25 mg of each catalyst (totally 50 mg of the blended catalyst) was used for 12 h reaction. 50 μL of each arene (1–5) corresponds to 541, 490, 485, 470, and 548 μmol, respectively.

The scope of the substituted arenes was examined for the photocatalytic direct DCC reaction with THF by using different catalysts. The results are shown in Tables S1–S4 in the ESI.†Fig. 7 shows the representative results of photocatalytic direct DCC between different arenes (1–5) and THF with the blended catalyst consisting of a TiO_2_ photocatalyst and an Al_2_O_3_ supported Pd–Au bimetallic catalyst. Substituted benzenes with electron withdrawing (2 and 3) and electron releasing (4 and 5) substituents successfully underwent the DCC reaction with THF. Benzaldehyde (2) and a benzonitrile (3) noticeably gave appreciable yields of the DCC products with high selectivity. The details such as the regioselectivity of the DCC products have not been fully clarified yet since the syntheses of the reference products are not easy by the conventional methods. In other words, these results show the importance of the further development of these new catalyses for the photocatalytic DCC reactions.

Benzaldehyde (2) and THF reacted to give a mixture of ortho, meta, and para substituted products (collectively shown as 2a) along with the oxidation and reduction products of 2, *i.e.*, benzoic acid (2b) and benzyl alcohol (2c), respectively, as undesired by-products (Table S1†). As mentioned in the ESI,† benzoic acid (2b) was the major product of this reaction, which resulted in a low selectivity to 2a, the DCC products. The autoxidation of benzaldehyde (2) to benzoic acid (2a) is well known and the presence of adsorbed water on the TiO_2_ photocatalyst might promote this reaction.^16^ Although both the pristine TiO_2_ and Pd(3.0)/TiO_2_ samples showed low selectivity to 2a (Table S1,† entries 1 and 2), the blended catalyst consisting of the TiO_2_ photocatalyst and the bimetallic Pd(2.0)Au(1.0)/Al_2_O_3_ catalyst slightly increased the formation of the DCC products and suppressed the formation of the side products, which improved the selectivity to 2a (Table S1,† entry 5). Change in the reaction conditions resulted in a high selectivity of 75% (Fig. 7, 2a).

The reaction between benzonitrile (3) and THF also gave a mixture of three DCC products (collectively shown as 3a), along with benzamide (3b) (Table S2†). The blended catalyst with the bimetallic catalyst again exhibited the highest activity for the formation of the DCC products (Table S2,† entry 5), although the selectivity was lower than the photocatalyst alone due to the increased production of 3b (Table S2,† entry 3). It is notable that, however, the improved reaction condition resulted in almost complete DCC selectivity (% *S* > 99, Fig. 7, 3a).

The photocatalytic direct DCC reaction of toluene (4) and aniline (5) with THF also proceeded (Tables S3 and S4†). Both reactions gave a mixture of the corresponding DCC product isomers, 4a and 5a, along with some side products. Photocatalytic oxidation of toluene (4) by adsorbed water to benzaldehyde (4b) and benzyl alcohol (4c) was a competitive reaction to the DCC to give 4a. The blended catalyst with the bimetallic catalyst drastically improved the selectivity to the DCC products (Table S3,† entry 5). Under the optimised conditions shown in Fig. 7, 90% selectivity to the DCC products 4a was obtained for toluene. The reaction between aniline (5) and THF, on the contrary, yielded DCC products with poor selectivity. Due to the high reactivity of the NH_2_ group of aniline (5), the homocoupling of aniline to give di(phenyl)diazine (5b) was the main reaction rather than the DCC between aniline and THF (Table S4†).^17^ NH_2_ group would be readily oxidised by the photogenerated holes on TiO_2_, which is suggested from the fact that ammonia can be easily oxidised to become amide radicals.^18^ This resulted in the very low selectivity to the DCC products, 5a, even under the optimised conditions (Fig. 7).

Table 3 shows the comparison of the total yield (%) of the DCC products (μmol) and selectivity (%, in the parenthesis) for the reaction using above-mentioned substrates (1–5). For the arene without any substituent (1) and the ones with electron withdrawing substituents like CHO (2) and CN (3), the blended catalyst consisting of the TiO_2_ photocatalyst and the bimetallic Pd(2.0)Au(1.0)/Al_2_O_3_ catalyst exhibited a higher activity to form the corresponding DCC products than the metal loaded TiO_2_ photocatalysts (Pd(3.0)/TiO_2_ and Pd(2.0)Au(1.0)/TiO_2_) and the blended catalyst with the monometallic catalyst (TiO_2_ + Pd(3.0)/Al_2_O_3_) (Table 3, entries 1–3). So, the new blended catalyst, with the bimetallic catalyst, developed in this study is the most selective and active catalyst for the photocatalytic direct DCC reactions between these arenes and THF. Also, the lower reactivity of the arenes with electron releasing substituents (4 and 5) (Table 3, entries 4 and 5) indicates that an electron deficient arene is more reactive towards the cross-coupling reaction than an electron rich arene. These results suggest that the photogenerated THF radical species is nucleophilic, so that it would prefer to react with an electron deficient aromatic ring. This property of THF radical is opposite to a photocatalytically generated electrophilic OH radical observed in our previous study.^17*a*^ These results are consistent with the fact that the electron deficient Pd species in the Pd–Au bimetallic catalyst is more active than pure Pd metal catalyst for the DCC reactions. This electron deficient Pd species can withdraw the electron density from an adsorbed arene molecule to accelerate its reaction with the nucleophilic THF radical.

**Table 3:** Comparison of the results of the photocatalytic direct DCC between various arenes and THF with the photocatalysts and the blended catalysts^a^

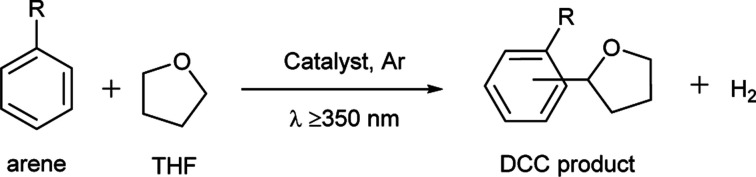						
Entry	Arene	DCC products	Yield of DCC products, *Y* (%)^b^			
			Pd(3.0)/TiO_2_	Pd(2.0)Au(1.0)/TiO_2_	TiO_2_ + Pd(3.0)/Al_2_O_3_	TiO_2_ + Pd(2.0)Au(1.0)/Al_2_O_3_
1^c^	1	1a	0.04(>99)	0.006(>99)	0.05(>99)	0.06(>99)
2^d^	2	2a	1.24(36)	0.42(32)	1.06(43)	1.73(46)
3^e^	3	3a	0.10(80)	0.08(82)	0.06(68)	0.27(57)
4^f^	4	4a	0.05(64)	0.02(74)	0.01(50)	0.03(90)
5^g^	5	5a	0.02(19)	0.003(6)	0.02(14)	0.02(23)

a 2 mL (19–24 mmol) of arene, 2 mL (24 mmol) of THF, and 50 mg of the photocatalyst or 100 mg of the blended catalyst were used, other conditions were the same as those described in Table 1.

b The total yield, *Y* (%), of DCC products. The value in parenthesis shows % *S*. The definition of % *Y* and % *S* is mentioned in the Experimental section.

c 2 mL (22.4 mmol) of benzene (1).

d 2 mL (19.9 mmol) of benzaldehyde (2).

e 2 mL (19.4 mmol) of benzonitrile (3).

f 2 mL (19.01 mmol) of toluene (4).

g 2 mL (21.9 mmol) of aniline (5).

### Proposed reaction mechanism for the photocatalytic direct DCC between arenes and THF

**Fig. 8:**
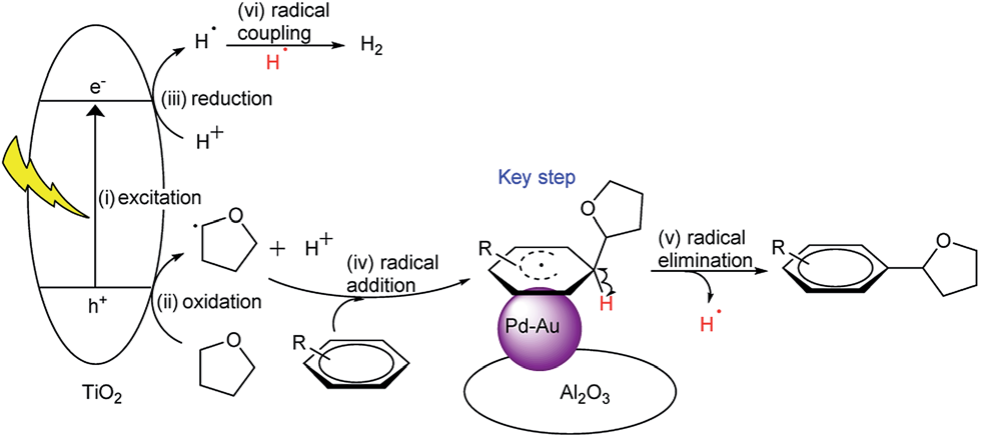
Proposed mechanism for the photocatalytic direct DCC between arenes and THF by the blended catalyst consisting of a TiO_2_ photocatalyst and a supported Pd–Au bimetallic catalyst.

Based on the mechanism proposed in the previous study^3*c*^ and the above results, the following reaction mechanism can be proposed for the photocatalytic direct DCC reaction between arenes and THF with the blended catalyst, which is a hybrid of TiO_2_ photocatalysis and metal catalysis by Pd–Au bimetallic nanoparticles (Fig. 8). The photo-excitation of TiO_2_ generates electrons and holes in the conduction band and the valence band, respectively (i). The photogenerated hole oxidizes the THF molecule to form a radical species and proton (ii). The proton is reduced to hydrogen radical by the photoexcited electron on TiO_2_ (iii). The THF radical can migrate to the bimetallic catalyst and attack to the carbon in the aromatic ring of the activated arene molecule, adsorbed on the Pd–Au bimetallic catalyst (iv), which gives the DCC product with the elimination of a hydrogen radical (v). The two hydrogen radicals can combine to give a hydrogen molecule either on the TiO_2_ surface or on the bimetallic catalyst (vi).

As mentioned before, electron deficient arenes exhibited the highest activity for this reaction. So, the nature of Pd catalysis in this photocatalytic reaction is proposed to be the activation of the aromatic ring by withdrawing its electron density, which facilitates its reaction with a photogenerated THF radical. The presence of an electron deficient Pd species, formed by the introduction of Au, would promote this reaction.

According to this mechanism for the DCC, it is also proposed that the THF radical species, photocatalytically formed on the TiO_2_ surface, can migrate to the separated metal catalyst. This suggests that the life of the THF radical species is not so short in these condition. Additionally, the coupling of two THF radical species can give the homocoupling products like octahydro-2,2′-bifuran.^3*e*^ But, as results show, this was a very minor reaction, meaning that the THF radical would selectively react with the activated arene molecule to give the DCC products.

## Conclusions

In the present work, we found that the photocatalytic direct DCC between various arenes and THF was a hybrid of TiO_2_ photocatalysis and Pd metal catalysis. The Pd metal catalyst can work even if they were on a photo-inactive support like Al_2_O_3_. Based on these findings, we developed a novel blended catalyst consisting of a TiO_2_ photocatalyst and an Al_2_O_3_ supported Pd–Au bimetallic catalyst that exhibited higher activity and selectivity for the photocatalytic direct DCC between arenes and THF than the conventional metal loaded TiO_2_ photocatalyst alone. Although the reported yields are still low, this work has provided some new insights about the mechanisms of the photocatalytic organic synthesis reactions and opened new prospects for the catalyst design. This blended catalyst has wide flexibility for modification, like, the photocatalyst part by using a co-catalyst or by changing the photocatalyst itself, the metal catalyst part by changing the loading amount, dispersion, or the metal component, and also the ratio of the two catalytic components. Thus, the blended catalyst can be further improved and become available for other photocatalytic direct DCC reactions and also other reaction systems.

## Conflicts of interest

There are no conflicts to declare.

## Supplementary Material

RA-008-C8RA02948B-s001
