# Supplementary material for: Novel blended catalysts consisting of a TiO2 photocatalyst and an Al2O3 supported Pd–Au bimetallic catalyst for direct dehydrogenative cross-coupling between arenes and tetrahydrofuran
Source: RSC Adv. 2018 Jul 2;8(42):24021–8. doi: 10.1039/c8ra02948b (PMC9081731; doi:10.1039/c8ra02948b)
Supplement: RA-008-C8RA02948B-s001 [file RA-008-C8RA02948B-s001.pdf]

Electronic Supplementary Material (ESI) for RSC Advances.
This journal is © The Royal Society of Chemistry 2018

Electronic Supplementary Information

Novel blended catalysts consisting of TiO_2 photocatalyst and Al_2O_3 supported Pd-Au bimetallic catalyst for direct dehydrogenative cross-coupling between arenes and tetrahydrofuran

Akanksha Tyagi,[a] Akira Yamamoto [a, b] and Hisao Yoshida *[a, b]

[a] Graduate School of Human and Environmental Studies, Kyoto University, Yoshida Nihonmatsu-cho, Sakyo-ku, Kyoto 606-8501, Japan.

[b] Elements Strategy Initiative for Catalysts and Batteries (ESICB), Kyoto University, Kyotodaigaku-Katsura, Nishikyo-ku, Kyoto 615-8520, Japan.

*E-mail: yoshida.hisao.2a@kyoto-u.ac.jp

Table of Contents

1. Synthesis of 4-(tetrahydrofuran-2-yl)benzonitrile
2. Photocatalytic direct DCC between various arenes and THF

1. Synthesis of 4-(tetrahydrofuran-2-yl)benzonitrile

The compound 4-(tetrahydrofuran-2-yl)benzonitrile was synthesized by following a reported procedure with slight modifications.[S1] 4,4'-Di-tert-Butyl-2,2'-bipyridine (13.8 mg, 0.0513 mmol) and $NiNO_3 \cdot 6H_2O$ (9.9 mg, 0.054 mmol) were put in a 100 mL round bottom flask, and placed in argon. Next, 3 mL of dry, degassed THF was added and the resultant mixture was stirred for 5 h at 323 K. This step was followed by the addition of another 18 mL of THF, bromobenzonitrile (0.35 mmol, 1 equiv), $Ir[dFCF_3ppy]_2(bpy) \cdot PF_6$ (10.3 mg, 6 mol%, 0.06 mmol), 4,4'-dimethoxybenzophenone (63 mg, 75 mol %, 0.26 mmol), and K_2HPO_4 (366 mg, 6.0 equiv, 2.1 mmol). The resultant mixture was stirred for 24 h, under the light irradiation from a xenon lamp with constant cooling by a fan, to control the reactor's temperature. After 24 h, the crude reaction mixture was filtered by a cylindrical plug of Celite with thorough rinsing with CH_2Cl_2 and EtOAc (10–20 mL). The filtrate was concentrated by rotary evaporation. The pure product was obtained by a silica gel column chromatography of the above filtrate, with an EtOAc–hexane mixture. The product was obtained as a colorless oil. ^1H NMR ($CDCl_3$, 400 MHz): δ = 7.63 (d, 2H), 7.42 (d, 2H), 4.93 (t, 1H), 4.09 (q, 1H), 3.96 (q, 1H), 2.37 (m, 1H), 2.01 (m, 2H), 1.739 (m, 1H). The NMR data matched with those in literature.[S1]

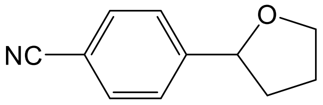

4-(tetrahydrofuran-2-yl)benzonitrile

2. Photocatalytic direct DCC between various arenes and THF

Various substituted arenes, with both electron withdrawing and electron releasing groups, were examined for the photocatalytic direct dehydrogenative cross-coupling (the photocatalytic direct DCC) with THF, as mentioned in the main text. All arenes successfully underwent the cross-coupling reaction to give the corresponding DCC products and hydrogen gas, as evidenced by the GC-TCD chromatogram in Fig. S1. Further, for all reactions, the blended catalyst containing the TiO_2 photocatalyst and the $Pd(2.0)Au(1.0)/Al_2O_3$ catalyst gave the highest activity and/or selectivity for the DCC products in these conditions.

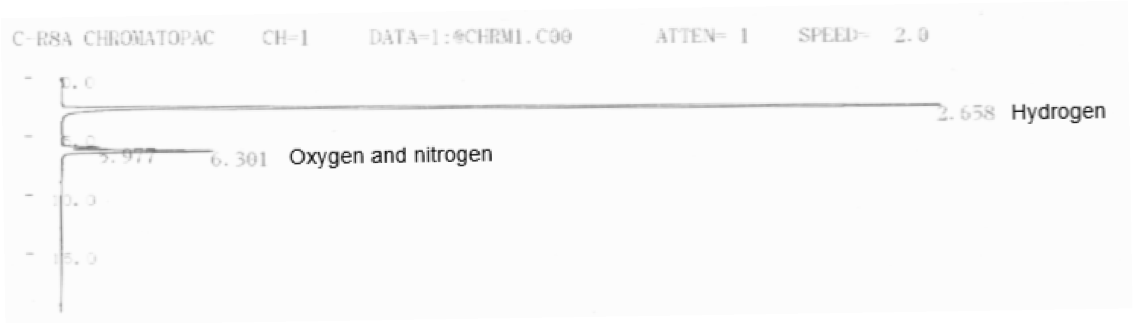

Fig. S1 The GC-TCD chromatogram of the photocatalytic direct DCC between benzene and THF with the $Pd(3.0)/TiO_2$ photocatalyst.

Table S1 shows the results of the photocatalytic direct DCC between benzaldehyde (**2**) and THF with different catalysts. This reaction gave a mixture of ortho, meta and para substituted products, collectively shown as **2a**, along with the oxidation and reduction products of benzaldehyde, benzoic acid (**2b**) and benzyl alcohol (**2c**) as by-products. Unfortunately, for all the catalysts tested for this reaction, the benzoic acid was the major product in these reaction conditions, which decreased the selectivity to the DCC product (Table S1, entries 1–6). This oxidation reaction is quite well known and the presence of the adsorbed water on the TiO_2 surface might facilitate this reaction.[S2] However, even in this condition, the blended catalyst containing the TiO_2 photocatalyst and the Pd(2.0)Au(1.0)/Al_2O_3 catalyst gave the highest activity and selectivity for the DCC products among the listed catalysts. The better reaction condition provided higher selectivity as shown in Fig. 7 in the main text.

Table S1 Photocatalytic direct DCC between benzaldehyde and THF with different catalysts [a]

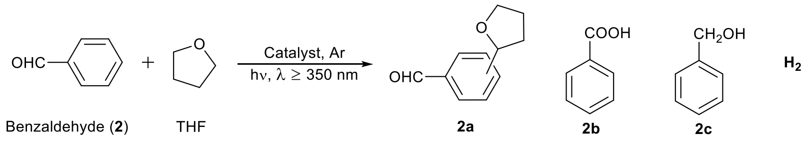

Entry	Catalyst	**2a** (μmol) [b]	**2b** (μmol) [b]	**2c** (μmol) [b]	%S [c]
1	TiO_2	151.8	292.5	55.6	37
2	Pd(3.0)/TiO_2	246.6	400.2	29.6	36
3	Pd(2.0)Au(1.0)/TiO_2	83.6	164.1	7.6	32
4	TiO_2 + Pd(3.0)/Al_2O_3	211.2	333.3	46.1	43
5	TiO_2 + Pd(2.0)Au(1.0)/Al_2O_3	344.8	359.8	49.7	46
6	TiO_2 + Au(3.0)/Al_2O_3	166.6	283.3	74.3	38

[a] General reaction conditions: 2 mL of arene, here benzaldehyde (**2**) (19.9 mmol), 2 mL (24 mmol) THF, and 50 mg of each catalyst was used for the reaction. The wavelength (λ) of the irradiated light was ≥ 350 nm, the light intensity was 40 mW/cm^2 (measured at 360 nm±60 nm). [b] The amount of all products was approximately determined from the calibration curve of 4-(tetrahydrofuran-2-yl)benzonitrile. [c] Selectivity for DCC products based on benzaldehyde, calculated as: %S=100×**2a** (μmol)/[**2a**+**2b**+**2c** (μmol)].

Table S2 shows the results of the photocatalytic DCC reaction between benzonitrile (**3**) and THF carried out with different catalysts. The reaction gave a mixture of three DCC products namely, 2-(tetrahydrofuran-2-yl)benzonitrile, 3-(tetrahydrofuran-2-yl)benzonitrile, and 4-(tetrahydrofuran-2-yl)benzonitrile (shown collectively as **3a**), and the oxidation product of **3**, benzamide (**3b**) (Table S2, entries 1–6). The blended catalyst consisting of the TiO_2 photocatalyst and the Pd(2.0)Au(1.0)/Al_2O_3 catalyst gave the highest yield of the DCC product, albeit with low selectivity (Table S2, entry 5). However, under the improved reaction conditions, as shown in Fig. 7 in the main text, the oxidation of **3** could be suppressed to get complete selectivity to the DCC products was achieved.

Table S3 shows the results of the photocatalytic DCC between toluene (**4**) and THF carried out with different catalysts. This reaction gave a mixture of two cross-coupling products namely 2-(o-tolyl)tetrahydrofuran and 2-(p-tolyl)tetrahydrofuran (shown collectively as **4a**), along with the oxidation products from toluene like benzaldehyde (**4b**) and benzyl alcohol (**4b**). The oxygen, for the formation of these oxidation products, would originate from the adsorbed water or surface hydroxyl groups on the catalysts. The partial oxidation of toluene to products like benzaldehyde is extensively reported in the literature.[S3] Only the oxidation of toluene proceeded with the pristine TiO_2 photocatalyst (Table S3, entry 1). However, the introduction of the Pd metal nanoparticles in the reaction mixture, supported on TiO_2 or Al_2O_3, gave the DCC product (Table S3, entries 2–6). The

blended catalyst consisting of the TiO_2 photocatalyst and the Pd(2.0)Au(1.0)/Al_2O_3 catalyst gave a moderate yield of the DCC product with the highest selectivity (Table S3, entry 5).

Table S2 Photocatalytic direct DCC between benzonitrile and THF with different catalysts [a]

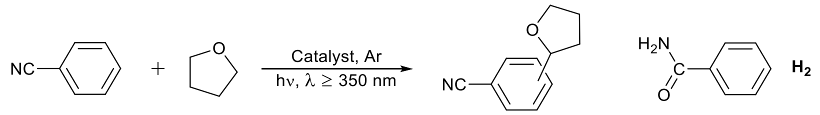

Benzonitrile (**3**) THF **3a** **3b**

Entry	Catalyst	**3a** (µmol) [b]	**3b**(µmol) [b]	**%S**
1	TiO_2	6.1	0.9	87
2	Pd(3.0)/TiO_2	19.3	4.8	80
3	Pd(2.0)Au(1.0)/TiO_2	16.2	3.4	82
4	TiO_2 + Pd(3.0)/Al_2O_3	10.9	5.1	68
5	TiO_2 + Pd(2.0)Au(1.0)/Al_2O_3	52.1	39.7	57
6	TiO_2 + Au(3.0)/Al_2O_3	12.5	3.6	78

[a] 19.4 mmol of benzonitrile (**3**), other reaction conditions were same as those in Table S1. [b] The amount of all products was approximately determined from the calibration curve of 4-(tetrahydrofuran-2-yl)benzonitrile.

Table S3 Photocatalytic direct DCC between benzonitrile and THF with different catalysts [a]

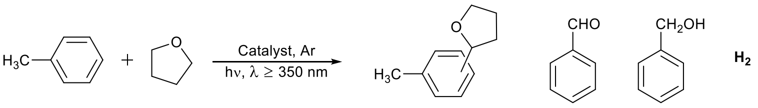

Toluene (**4**) THF **4a** **4b** **4c**

Entry	Catalyst	**4a** (µmol) [b]	**4b** + **4c** [b]	**%S**
1	TiO_2	0.0	9.9	0.0
2	Pd(3.0)/TiO_2	10.3	5.9	64
3	Pd(2.0)Au(1.0)/TiO_2	4.7	1.6	74
4	TiO_2 + Pd(3.0)/Al_2O_3	2.8	2.8	50
5	TiO_2 + Pd(2.0)Au(1.0)/Al_2O_3	5.2	0.6	90
6	TiO_2 + Au(3.0)/Al_2O_3	0.0	0.0	0

[a] 19.01 mmol of toluene (**4**), other reaction conditions were the same as those in Table S1. [b] The amount of all products was approximately determined from the calibration curve of 4-(tetrahydrofuran-2-yl)benzonitrile.

The photocatalytic DCC reaction between aniline (**5**) and THF also gave a mixture of two products namely, 2-(tetrahydrofuran-2-yl)aniline and 4-(tetrahydrofuran-2-yl)aniline (shown collectively as **5a**) and the homocoupling products from aniline, di(phenyl)diazine (**5b**). Table S4 shows the results of the reaction carried out with different catalysts. The NH_2 group of aniline was very reactive in the photocatalytic reaction, which promoted its homocoupling to give **5b.** This reaction mainly occurred for this system, which decreased the selectivity to DCC products (Table S4, entries 1–6). Even then, the blended catalyst consisting of the TiO_2 photocatalyst and the Pd(2.0)Au(1.0)/Al_2O_3 catalyst gave the highest yield of the DCC product with the highest selectivity (Table S4, entry 5).

Table S4 Photocatalytic direct DCC between aniline and THF with different catalysts [a]

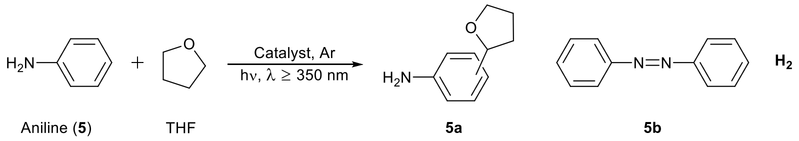

Entry	Catalyst	**5a** (μmol) [b]	**5b** (μmol) [b]	%S
1	TiO_2	4.2	14.9	22
2	Pd(3.0)/TiO_2	5.2	22.1	19
3	Pd(2.0)Au(1.0)/TiO_2	0.6	10.5	6
4	TiO_2 + Pd(3.0)/Al_2O_3	3.4	20.2	14
5	TiO_2 + Pd(2.0)Au(1.0)/Al_2O_3	5.2	17.6	23
6	TiO_2 + Au(3.0)/Al_2O_3	3.7	34.2	10

[a] 21.9 mmol of aniline (**5**), other reaction conditions were the same as those in Table S1. [b] The amount of all products was approximately determined from the calibration curve of 4-(tetrahydrofuran-2-yl)benzonitrile.

References

S1. D. R. Heitz, J. C. Tellis and G. A. Molander, *J. Am. Chem. Soc.*, 2016, **138**, 12715.

S2. a) M. Conte, H. Miyamura, S. Kobayashi and V. Chechik, *Chem. Commun.*, 2010, **46**, 145; b) M. Sankar, E. Nowicka, E. Carter, D. M. Murphy, D. W. Knight, D. Bethell and G. J. Hutchings, *Nat. Commun.*, 2014, **5**, 3332

S3. E. J. Park, H. O. Seo and Y. D. Kim, *Catal. Today*, 2017, **295**, 3.
